# Supplementary material for: The genome sequence of a click beetle, Limonius poneli Leseigneur & Mertlik, 2007 (Coleoptera: Elateridae)
Source: Wellcome Open Res. 2026 Apr 30;11:251. [Version 1] doi: 10.12688/wellcomeopenres.26311.1 (PMC13305553; doi:10.12688/wellcomeopenres.26311.1)
Supplement: Peer review history

# Reviewer report: Reviewer response for version 1

**DOI:** 10.21956/wellcomeopenres.28976.r155745

**Published:** 2026-06-25

**Authors:** Christopher B. Cunningham

**Affiliations:** 1 University of Georgia, Atlanta, Georgia, USA

This is a report of the genome assembly of the click beetle *Limonius poneli*. I think this is a good resource for the insect genomics community. The methods are sound and relatively well documented. The article needs a few more details to be reasonably reproducible. A genome assembly without annotation is of reduced use to the community.

 Abstract. Bare bones report of assembly length. No estimates of the genome (% assembled of estimated size) or its gene compliment completeness (BUSCO of genome and annotation) are given.

 Keywords. Should be alphabetical order. Nothing from the title needs to be repeated.   

 Background. Almost all the details of its life history are irrelevant to the reader. The paper is not about its ecology or morphology, but about its genetics. The reader does not really understand that from this background. Are there close relatives that have been sequenced? Does it serve as a model for any physiology. Direct mention the motivation is that it is part of the Darwin Tree of Life project.

 Figure 2/3/4/5 titles and description in manuscript. The paragraph of Fig 2/3 should say the results, not what the figure contains – that should be found in the caption. The titles should be informative and tell the reader what you would like them to understand about the data; e.g., Fig 3/4. Little contamination was found in the final genome assembly.

 Figure 4. Fig 4 has very little information beyond what is found elsewhere in the manuscript and can easily be summarized as a sentence in the results.

 All parameter setting if they were different from default of each piece of bioinformatic software needs to be specified. Just put a sentence to begin that everything was default unless specified otherwise. What assessment was done to ensure that default was adequate? I assume none as this is a large project, but the reader should be told that.

 Table 4 repeats much of what was already stated in the restuls. Table 5 is of little added value. Just add the version numbers of software used inline at the appropriate places in the methods section.

 Is the Ethics statement needed? It just says the provider of the sample should meet some agreed upon standard, but does not actually say that they did in this case. Either drop the statement or actually say the standard was met for this sample. This is not a paper about the standards of collection.

 Add something to the report that contextualize the research for the reader. Is this species the first of a taxon to be assembled, is it an outgroup to some established model species, is it now possible to investigate some interesting aspect of the species biology, etc? This does not to be extensive or highly directed, but there is currently no justification of this work outside of the Data Availability Statement.

Are sufficient details of methods and materials provided to allow replication by others?

Yes

Is the rationale for creating the dataset(s) clearly described?

Yes

Are the datasets clearly presented in a useable and accessible format?

Yes

Are the protocols appropriate and is the work technically sound?

Yes

Reviewer Expertise:

Insects. Genomics. Genetics. Epigenetics. Behavior. Reproduction.

I confirm that I have read this submission and believe that I have an appropriate level of expertise to confirm that it is of an acceptable scientific standard.

# Reviewer report: Reviewer response for version 1

**DOI:** 10.21956/wellcomeopenres.28976.r155749

**Published:** 2026-06-01

**Authors:** Panagiotis Ioannidis

**Affiliations:** 1 Foundation for Research & Technology - Hellas, Crete, Greece

This paper presents the genome sequencing and assembly of a click beetle; Limonius poneli.

 The assembly is of high quality with >99% of the assembled sequence assigned to one of 10 chromosome-level scaffolds. There are quite a few additional scaffolds, but this is okay, especially given the large size of the genome (>1 Gbp). QV and k-mer completeness are also really good, as are BUSCO scores.

 I find the blob plot (figure 6) particularly interesting, because it shows that some of the "un-anchored" scaffolds form a cluster with very low GC content (centered around ~10%). And since the coverage is very similar to that of the chromosome-level scaffolds, it seems that they are part of the nuclear genome (and not bacterial symbionts, for example). Could the authors comment on this finding? And also could they comment on the other, higher-GC cluster of un-anchored scaffolds (around 52%)?

 Generally speaking, the authors should be highlighting a few of the findings of their analysis. I'm not saying that they should write extensive texts. I understand that this is a genome report. However, they should, at least, write a couple of sentences for things that stand out in a figure, or for something of particular importance (e.g. a relatively large genome).

 Lastly, I was glad to see that the genome will be annotated, even though it's currently not annotated. Have the authors also generated RNAseq data from the same sample they used for genome sequencing? Because they mention that "The genome will be annotated using available RNA-seq data...", but it's not clear the origin of this "available" RNAseq data.

 Minor significance:

 - The full binomial name (Limonius poneli) should only be written once. After that, it should be referred to as "L. poneli".

 - Why don't the authors use some kind of thousands separator? They currently use a space (e.g. they write "1 286.06 Mbp") but this is confusing. It would be much better if they used for example a comma "," instead.

Are sufficient details of methods and materials provided to allow replication by others?

Yes

Is the rationale for creating the dataset(s) clearly described?

Yes

Are the datasets clearly presented in a useable and accessible format?

Yes

Are the protocols appropriate and is the work technically sound?

Yes

Reviewer Expertise:

insect genomics, bioinformatics

I confirm that I have read this submission and believe that I have an appropriate level of expertise to confirm that it is of an acceptable scientific standard.

# Reviewer report: Reviewer response for version 1

**DOI:** 10.21956/wellcomeopenres.28976.r154923

**Published:** 2026-05-07

**Authors:** Rajib Deb

**Affiliations:** 1 ICAR-National Research Centre, Guwahat, Assam, India

The manuscript demonstrates strong technical quality and presents a valuable genome dataset; however, several aspects limit full reproducibility. The methods rely heavily on external protocols without summarizing key parameters within the text, making it harder for readers to independently replicate the workflow. Additionally, important tool settings (e.g., for Hifiasm, YaHS, and Merqury) are not explicitly reported. The manual curation process is only briefly referenced and lacks sufficient detail on decision criteria. Finally, the absence of a clear, consolidated workflow diagram reduces overall clarity. Addressing these issues would significantly improve transparency and usability.

Are sufficient details of methods and materials provided to allow replication by others?

Partly

Is the rationale for creating the dataset(s) clearly described?

Yes

Are the datasets clearly presented in a useable and accessible format?

Yes

Are the protocols appropriate and is the work technically sound?

Yes

Reviewer Expertise:

Genomics

I confirm that I have read this submission and believe that I have an appropriate level of expertise to confirm that it is of an acceptable scientific standard, however I have significant reservations, as outlined above.
